# Early Social Behavior in Young Children with Sex Chromosome Trisomies (XXX, XXY, XYY): Profiles of Observed Social Interactions and Social Impairments Associated with Autism Spectrum Disorder (ASD)

**DOI:** 10.1007/s10803-022-05553-8

**Published:** 2022-05-12

**Authors:** Nienke Bouw, Hanna Swaab, Nicole Tartaglia, Lisa Cordeiro, Sophie van Rijn

**Affiliations:** 1 Clinical Neurodevelopmental Sciences, Leiden University, Leiden, The Netherlands; 2 Leiden Institute for Brain and Cognition, Leiden, The Netherlands; 3 Developmental Pediatrics, Children’s Hospital Colorado, University of Colorado School of Medicine, Aurora, CO USA; 4 Department of Pediatrics, University of Colorado School of Medicine, Aurora, CO USA; 5 Clinical Neurodevelopmental Sciences, Faculty of Social and Behavioral Sciences, Leiden University, PO Box 9500, 2300 RA Leiden, The Netherlands

**Keywords:** Sex Chromosome Trisomies, Social behavior, Autism Spectrum Disorder, Klinefelter syndrome, Trisomy, Triple X

## Abstract

Individuals with Sex Chromosome Trisomies (SCT; XXX, XXY, XYY) have an increased vulnerability for developing challenges in social adaptive functioning. The present study investigates social interaction behavior in the context of varying social load, and Autism Spectrum Disorder (ASD) symptomatology in young children aged 1–7.5 years old, with SCT (*N* = 105) and control children (*N* = 101). Children with SCT show less interaction behaviors and more social withdrawal, as compared to their control peers, which were most evident in the high social load condition. Second, social impairments related to ASD are more prevalent, as compared to controls (27.1% at clinical level). These findings stress the importance of early monitoring and (preventive) support of early social development in young children with SCT.

Approximately 1–650 to 1–1000 children is born with a Sex Chromosome Trisomy (SCT; Boyd et al., 2011). SCT, the presence of an extra X- or Y-chromosome, lead to the chromosomal patterns of 47, XXX in girls (Triple/Trisomy X), and 47, XXY (Klinefelter’s syndrome) and 47, XYY (XYY syndrome) in boys. SCT has been associated with a mild physical phenotype shared across SCT conditions, such as mild facial characteristics, a tall stature, and low muscle tone (Tartaglia et al., 2020). Cognitive functioning in SCT is within normal limits, although somewhat lower than average, specifically with respect to the language domain (see for a review: Leggett et al., 2010). Specific effects of the extra X- and Y-chromosome on neurobehavioral development have been found in self-regulation and social adaptation (Tartaglia et al., 2020; Urbanus, Swaab, et al., 2020; Urbanus, van Rijn, et al., 2020; van Rijn, 2019). SCT is consequently associated with increased risk for symptoms of social difficulties and Autism Spectrum Disorders (van Rijn, 2019).

To understand the pathways to impairment of social adaptation it is important to be aware that the presence of an additional X- or Y-chromosome is known to convergently impact the maturation of brain functions and networks involved in social adaptive cognitive and behavioral development (Hong & Reiss, 2014; Raznahan et al., 2016). Social cognition involves the abilities that enable us to understand social information and to interact with the social environment (Beauchamp & Anderson, 2010), and are central to interpersonal communication, to the development and maintenance of satisfying relationships with others (Rao et al., 2008), and are associated with quality of life (De Vries & Geurts, 2015). Since social adaptive development is anchored in early brain maturation and because the first years of life are important for rapid maturation and specialization of the social brain network, this period may mark a key period for the development of appropriate processing of social information, allowing successful social interaction with others (Grossmann & Johnson, 2007). It is therefore especially important to investigate the impact of SCT on social cognitive and social behavior during the early stages of childhood, to identify early markers of an ‘at risk’ social development. The current study was designed to investigate early social behaviors with a focus on provide insights into that, and focuses on the early impact of SCT on social interaction behaviors and risk on social impairments related to ASD in children aged 1–7.5 years.

Although individuals with SCT have unique and varying developmental profiles, there is sufficient evidence that, on average, the social behavioral presentation of SCT is characterized by challenges in social functioning. School-aged children, adolescents and adults with SCT may show shy, withdrawn and anxious social behavior, as well as difficulty with forming personal relationships, and with taking initiative in social contact (Bender et al., 1999; Otter et al., 2010; Ross et al., 2012; van Rijn et al., 2014). The outcomes of these studies suggest that individuals with SCT from school age on are at risk of experiencing difficulties in social adaptive functioning. However, research on social development before the age of six years is extremely limited. In order to sensitively and objectively explore the impact of SCT on social functioning early in development, we studied social interaction behaviors of young children with SCT during structured behavior observations. Increasing social interaction skills are necessary for developing the capacity to deal with more complex social information with more social information and a higher pressure to react in a social adaptive way (Soto-Icaza et al., 2015). We were therefore interested whether and how young children with SCT shape their social interaction behavior under varying levels of social load during social interactions. We manipulated the social load factor during the social interaction observation from provided social interactions with a low social load, in which the adult provided a play example for the child to follow but did not maintain the interaction, to a social interaction with no social load in which the adult remains passive in which it was up to the child to initiate and continue the interaction, and finally a social interaction with a high social load in which the adult gave the child directions and cues in a stable and high frequency which could lead to some minor stress in the child (Krug et al., 2008).

The severity of the impact of SCT on social development is illustrated by reports of increased risk for social impairments that are associated with ASD symptomatology in SCT. ASD is a clinical classification of neurobehavioral problems resulting in persistent deficits in social interaction and communication, and by repetitive behaviors and restricted interests (DSM; American Psychiatric Association, 2013). Compared to a worldwide prevalence rate of ASD of 0.6% in the general population (Elsabbagh et al., 2012), prevalence of ASD has been shown to be higher in SCT. On average across studies, depending on the ascertainment methods, diagnostic measurements and criteria used, 15% (range 10.8–20%) of individuals with 47, XXX meet full ASD criteria; 18% (range 10–27%) of individuals with 47, XXY and 30% (range 19–43%) of individuals with 47, XYY (see for a review: van Rijn, 2019). Rather than investigating the risk for ASD symptomatology as a categorical phenomenon, previous studies have also explored the impact of SCT on a range of social behavioral symptoms associated with ASD. These studies found high levels of ASD symptomology from school age onwards, with a relative strength with regard to social awareness and social motivation for boys and girls with an extra X-chromosome and a strength in social motivation for boys with 47, XYY (Cordeiro et al., 2012; Tartaglia et al., 2010; van Rijn et al., 2014; Wilson et al., 2019). However, these studies had broad age ranges from childhood to early adulthood, and did not investigate the impact of SCT on social impairments very early in life. To explore the extent to which early social vulnerabilities reflect high levels of symptoms that may belong to ASD, the present study investigates the impact of SCT on social impairments in the domains of social awareness, social cognition, social communication, social motivation and if there are restricted interests and repetitive behaviors during the first years of life.

Learning more about the early social development of children with SCT will shed light on early neurocognitive and neurobehavioral pathways to social challenges and related psychopathology later in life of children, adolescents and adults with SCT. Urgency for this knowledge is stressed by the increasing number of children diagnosed with SCT, as a result of advanced technology to screen for genetic variations before birth (i.e. NIPT; Samango‐Sprouse et al., 2017; Tartaglia et al., 2020). This growing group of prenatally diagnosed children with SCT also provides us with the unique opportunity to prospectively explore neurobehavioral profiles of a genetic at risk population, even before the behavioral phenotype of the genetic condition is revealed. That is to say, exploring early neurobehavioral profiles helps us to understand developmental pathways leading up to behavioral symptoms of SCT. In the literature, vulnerabilities in individuals with SCT are predominantly described in terms of global cognitive (i.e. intellectual functioning) and language deficits (Boada et al., 2009; Leggett et al., 2010; Ross et al., 2009), although recent studies also explored the impact of SCT on other neurocognitive outcomes, such as social cognition and executive functioning (see for reviews of findings in school age children and adolescents: van Rijn, 2019; Urbanus, Swaab, et al., 2020; Urbanus, van Rijn, et al., 2020). Although speculative, difficulties in coping with the social environment may not only be the result of a lower general understanding of the environment, but also lower levels of abilities to interact with the environment in terms of language and communication. A socially vulnerable developmental course may also be related to specific early impacts of SCT on social (cognitive) abilities such as being aware of social information, understanding social information, and shaping social interaction behaviors such as smiling, eye contact and imitation. Support for this hypothesis comes from recent studies that found an early impact of SCT on social cognition (Bouw et al., 2021), and social behavioral problems (Urbanus, Swaab, et al., 2020; Urbanus, van Rijn, et al., 2020). We therefore studied the role of global cognitive and language abilities in social adaptive behavior of children with SCT.

The current study aims to explore the early impact of SCT on profiles of social interaction behaviors under different levels of social load, and parent-reported social impairments associated with ASD in young children aged 1–7 years old, with regard to the type of vulnerability, the age dynamics and clinical severity of these behaviors. Second to these main research questions, we investigate the role of cognitive and language development on social outcomes, and to what degree the specific karyotypes (XXX vs. XXY vs. XYY) vary in risk for social vulnerabilities. Based on the relevance of the extra X- and Y- chromosome on brain networks that underlie the development of social adaptive behavior, and reported vulnerability for social difficulties in individuals with SCT, we hypothesized that on average young children with SCT might show less well developed social interaction abilities and elevated social impairments associated with ASD, as compared to a control sample. We also hypothesized a specific effect of SCT on social functioning, that is to say that the impact of SCT on social functioning is independent of global cognitive and language development.

## Methods

### Participants

The present study is part of a larger ongoing longitudinal study (the TRIXY Early Childhood Study—Leiden, The Netherlands), which includes children with SCT and nonclinical controls aged 1–7.5 years. The TRIXY Early Childhood Study aims to identify neurodevelopmental risk in young children with an extra X or Y chromosome.

A group of 105 children with SCT (range 1–7.5 years old; *M*_age_ = 3.66, *SD* = 1.94) was included in this study, as well as a population-based sample of 101 children (44 boys; *M*_age_ = 3.61, *SD* = 1.63). Mean age did not differ between groups (*t*(204) = 0.21, *p* = 0.837). The SCT group consisted of 33 girls with 47, XXX (31.4%), 50 boys with 47, XXY (47.6%) and 22 boys with 47, XYY (21.0%). Age did not differ between karyotypes (*F* (2,102) = 2.81, *p* = 0.065). Recruitment and assessment took place at two sites: the Trisomy of the X and Y chromosomes (TRIXY) Expert Center at Leiden University (LUBEC) in Leiden, The Netherlands, and the eXtraordinary Kids Clinic in Developmental Pediatrics at Children’s Hospital Colorado in the USA. Children in the SCT group were recruited in cooperation with the clinical genetics departments (from The Netherlands and Colorado, USA), as well as through patient-advocacy groups and social media postings. The diagnosis of SCT was defined by trisomy in at least 80% of the cells, which was confirmed by standard karyotyping. Seventy-one children (67.6%) were diagnosed prenatally (20 girls with XXX, 36 boys with XXY, 15 boys with XYY), and 34 children (32.4%); postnatally (13 girls with XXX, 14 boys with XXY, 7 boys with XYY). As ASD cannot be diagnosed prior to age 2, diagnosis status was available for 68% of the sample. Within this group, three parents reported that their child received a clinical diagnosis of ASD (1 boy with XXY, 2 boys with XYY).

For the SCT group, recruitment strategy was assessed, and three subgroups were identified: (1) ‘active prospective follow-up’, which included families who were actively followed after prenatal diagnosis (51.4% of the SCT group), (2) ‘Information seeking parents’, which included families who were actively looking for more information about SCT without having specific concerns about the behavior of their child (29.5% of the SCT group), and (3) ‘Clinically referred cases’, which included families seeking professional help based on specific concerns about their child’s development (19.0% of the SCT group). Twenty-four out of 50 boys with 47, XXY had received testosterone treatment (48.0%).

Control children were recruited from the western part of The Netherlands, and approached with information brochures about the study. All participants (child and parents) were Dutch or English speaking, had normal or corrected-to-normal vision, and did not have a history of traumatic brain injury. For ethical reasons, children in the control group were not subjected to genetic screening, as these children were meant to be a representation of the general population. As the prevalence of SCT is ~ 1 in 1000, the risk of having one or more children with SCT in the control group was considered minimal and acceptable.

### Measurements and Instruments

#### Structured Observations of Social Interactions: ASIEP-3

**Fig. 1:**
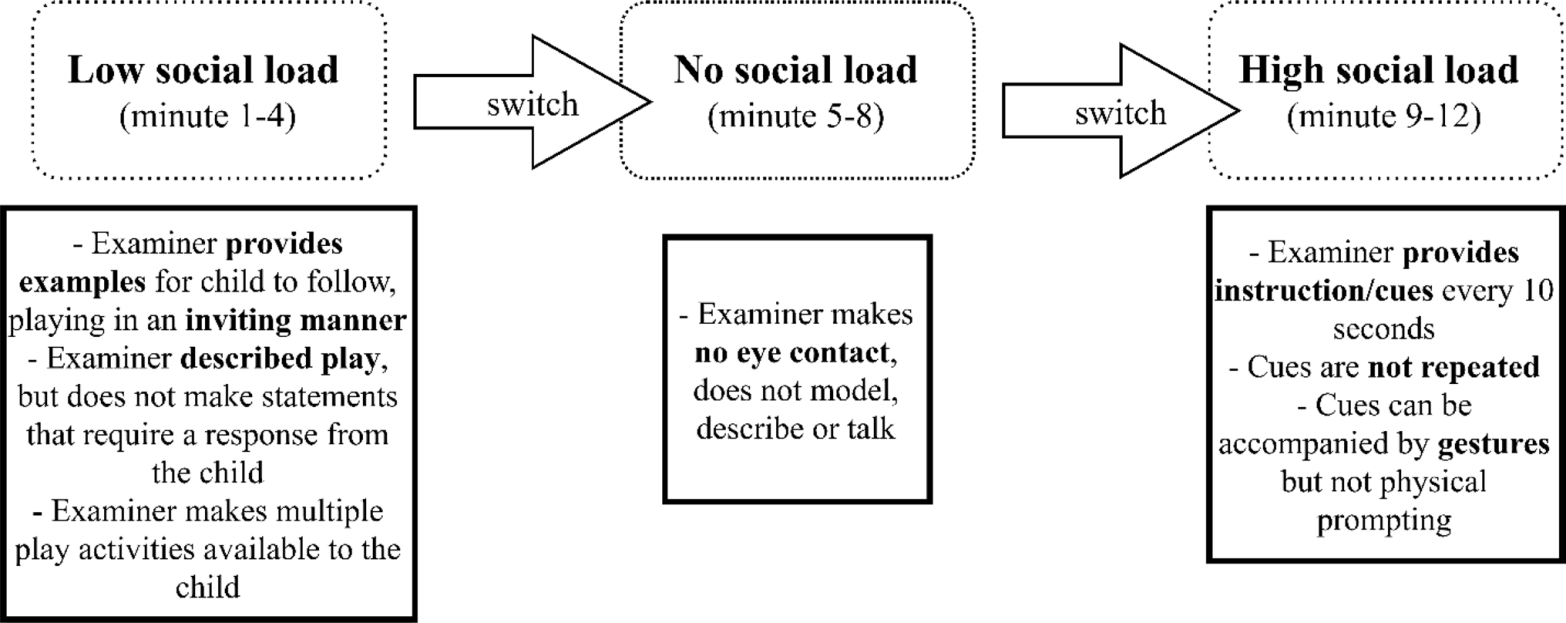
Administration of ASIEP-3, Interaction Assessment. Conditions of social load: administration and examples of statements ( adapted from Cordeiro et al., 2020)

The Interaction subtest of the Autism Screening Instrument for Educational Planning—Third Edition (ASIEP-3; Krug et al., 2008) was administered, in order to measure social interaction behaviors including spontaneous social responses, acknowledgement of direct requests, and the ability of the child to socially interact with an examiner under different conditions of external social load. The ASIEP-Interaction subtest is a time structured and standardized play setting, and consists of three different environmental conditions of four minutes each that were always administrated in the same order: (1) the active modeling condition: basic inviting parallel play modelled by the examiner, defined as the low social load condition, (2) the passive/no interaction condition: the withdrawal of engagement and attention by the examiner, defined as the no social load condition, and (3) the direct cues condition: the examiner gives specific cues and directions to the child, defined as the high social load condition. The examiner used age appropriate toys and activities during the play situation, as well as language appropriate for the skill level of the child. See Cordeiro et al., (2012) for additional descriptions of the interaction assessment, and Fig. 1 for an overview of the three conditions and task administration descriptions.

The ASIEP was video recorded and scored afterwards by trained independent raters, based on the procedures described by Krug et al., (2008). Raters were not involved in the assessment, and blind to the child’s group membership and karyotype. The videos were scored at 10-s intervals, and the observed behaviors were scored into one of four behavior codes: (1) Interaction, e.g. the child responds, initiates, touches or complies, (2) Constructive Independent Play, e.g. independent play without social interaction, (3) No Response, e.g. no observable behavior or response of the child, or self-stimulation and self-abuse, or (4) Aggressive behavior, e.g. tantrums, hits, cries, bites, etc.. The codes are summed yielding counts for each of these four behavior codes, within the three conditions (i.e. absent, low and high social load), and an overall total score (possible range 0–48).

#### Social Impairments Associated with Autism Spectrum Disorder (ASD): SRS-2

In order to measure ASD symptomology, the primary parent of the child completed the Social Responsiveness Scale—Second Edition (SRS-2; Constantino & Gruber, 2012). The SRS is a 65item parent-report questionnaire, designed to quantify ASD related social impairments. Dependent on the age of the child, two different versions of the SRS-2 were administrated to the primary caregiver of the child: the SRS—Preschooler version (children aged 3–4 years old) and the SRS—School age version (children aged 4–7.5 years old). Examples of items are: ‘Is able to understand the meaning of other people’s tone of voice and facial expressions’ and ‘Responds appropriately to mood changes in others (for example, when a friend’s or playmate’s mood changes from happy to sad)’. The parent rated the behavior of the child over the past six months on each item using a 4-point Likert scale ranging from 1 ‘not true’ to 4 ‘almost always true’, yielding a raw total score and gender-normed T-score on five subdomains of ASD symptoms (social awareness, social cognition, social communication, social motivation and restricted interests or repetitive behavior). Higher raw and T-scores indicate parental report of a higher and more severe presence of ASD symptoms. In addition to average behavioral outcomes on the five subdomains, we were also interested in percentages of children with SCT that had scores above clinical cut-off, indicating clinical risk of ASD. T-scores were used to calculate risk of ASD symptoms that are clinically relevant and associated with clinical diagnosis of ASD. T-scores between 65 and 75 correspond to a ‘moderate’ range of severity, and scores of 76 and higher are in the ‘severe’ range. The SRS-2 has strong internal consistency (Constantino & Gruber, 2012), and extensive proof of validity (Bruni, 2014).

#### Global Level of Cognitive and Language Development

In order to measure global level of intelligence, receptive and expressive language, developmental age appropriate instruments were used. The Bayley—Third Edition (cognitive, receptive language and expressive language scale; Bayley, 2006) was administered to 1–2 year old children. In the older children four subtests of the Wechsler Preschool and Primary Scales of Intelligence—Third Edition (WPPSI-III; Wechsler, 2002) were used to estimate global level of intelligence (children aged 3 years: Block Design, Receptive Vocabulary, Information, Object Assembly; children aged 4 years and older: Block Design, Matrix Reasoning, Vocabulary, and Similarities). For children aged 4 years and older, Total IQ estimates were calculated based on this short form version of the WPPSI-III (Hurks et al., 2016). The Peabody Picture Vocabulary Test—Third Edition (PPVT-III; Dunn & Dunn, 1997) was used to measure receptive language level in children aged 3 years and older. To assess expressive language skills, the Clinical Evaluation of Language Fundamentals—Preschool, Second Edition, was administrated to children of 3 years and older (CELF-Preschool; Wiig et al., 2004).

### Ethical Approval and Informed Consent

This study was approved by the Ethical Committee of Leiden University Medical Center, The Netherlands, and the Colorado Multiple Institutional Review Board (COMIRB) in Colorado, USA. Signed informed consent was obtained from the parents/guardians of all participating children, according to the declaration of Helsinki.

### Study Procedures

Assessment took place at various sites (Colorado (USA) and The Netherlands) either in a quiet room at the university or at home. To standardize the testing environment, the testing set-up and research protocols were identical for all sites. Researchers from Leiden University were responsible for project and data-management (i.e., training and supervision of researchers processing and scoring of data). Administration of the WPPSI-III, CELF-Preschool and PPVT-III was performed seated on a table by trained child psychologists in the Dutch or English language, depending on the first language of the child. The SRS-2 questionnaire was filled in by the primary caregiver of the child, either in Dutch or English. Administration of the ASIEP always took place after fixed amount of interaction time with the child before starting the test in order to prevent familiarity differences to interfere with the test scores.

### Statistical Analyses

Statistical Package for the Social Sciences (SPSS, version 25) was used for statistical analyses. Independent t-tests were used to test for differences between research sites. Repeated Measures ANOVAs were used to study differences in profiles of social interaction between the SCT and control group, in which the Greenhouse–Geisser correction was used if the assumption of sphericity was violated. To test for differences between the SCT and control group for social impairments, a MANOVA was carried out. Moderating effects of age on social interaction behavior and social impairments were tested with PROCESS analyses (Hayes, 2017). Pearson’s correlation were used to assess the association between social behavioral outcomes and cognitive abilities. When significant correlations were found, the cognitive parameter was added to the analyses as covariate (RM MANCOVA/MANCOVA). Two separate *t-*tests were used to measure SCT vs. control differences of social impairments in average and below-average IQ groups. Differences on social behavioral outcomes between karyotypes (XXX, XXY, XYY) and recruitment bias groups (active follow-up, information-seeking parents, clinically referred cases) were assessed and accounting for the effect of age with ANCOVAs. Statistical significance was set at *p* < 0.05 a priori. Statistical analyses were performed one-tailed (SCT vs. control) or two-tailed (moderating age effect, influence of karyotype, recruitment bias). Effect sizes were calculated with partial η^2^.

## Results

### Comparison Between Research Sites

No differences between research sites (The Netherlands, USA) were found for total score on the ASIEP-3 (*t* (87) = − 1.50, *p* = 0.138), and for total score on the SRS-2 (*t* (68) = − 0.38, *p* = 0.707). Therefore, all SCT data were collapsed across sites.

### Structured Observations of Social Interaction

#### Data Quality

The ASIEP-3 was successfully administrated and completed by 188 children enrolled in the study. 18 children were not able to complete the ASIEP administration, mainly due to non-compliance or unstandardized administration. Interrater reliability was calculated based on a randomly selected subsample of 10 participants, and showed an intraclass correlation coefficient (ICC) of 0.86—0.89 (for the ASIEP behavior codes collapsed together), which is considered excellent reliability (Cicchetti & Sparrow, 1981).

#### Social Interaction Behavior in SCT vs. Control

Over all ages, a significant difference between the SCT and control group was found for social interaction behavior, independent of social load condition (*F* (2.03, 376.97) = 9.17, *p* < 0.001, η_p_^2^ = 0.05). Pairwise comparisons revealed less Interaction behavior and more Constructive Independent Play in the SCT group as compared to control children, with medium effect sizes. No differences were found for No Response and Aggressive behaviors (see Table 1).

**Table 1:** Structured observations of social interactions in the SCT and control group: behavioral outcomes

	*N*	Missing	SCT		Control		SCT vs. control		
			Min–Max	*M (SD)*	Min–Max	*M (SD)*	*p*-value	Group differences	Effect-size (η_p_^2^)
Social interaction behavior	188	18							
Interaction			0–37	17.92 (7.86)	6–43	22.35 (8.39)	< .001	SCT < control	.07
Constructive independent play			0–41	21.66 (8.50)	1–39	17.24 (8.34)	< .001	SCT > control	.07
No response			0–41	.21 (8.69)	0–34	7.90 (7.19)	.786		
Aggression			0–4	.09 (0.47)	0–13	.22 (1.38)	.393		

*SCT* Sex Chromosome Trisomy, *ASIEP* Autism Screening Instrument for Education Planning

**Fig. 2:**
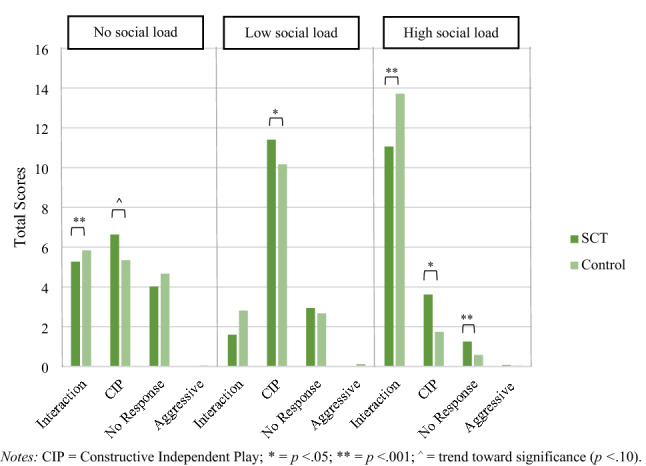
Social interactive behaviors (Interaction, CIP, No Response, Aggressive) under three conditions (no social load / low social load / high social load) in the SCT and control group. CIP = Constructive Independent Play; *p < .05; **p < .001; ^o^ = trend toward significance (*p* < .10)

To investigate the conditional effect of social load (no social load; low social load; high social load) on social interaction behavior between the SCT and control group, a RM ANOVA was used. A significant overall difference between the SCT and control group was found for social interaction behavior within the different conditions, *F* (3.98, 775.83) = 7.90, *p* < 0.001, η_p_^2^ = 0.04. In the no social load condition, as compared to controls the SCT group showed lower Interaction behaviors (*p* = 0.001), and a trend toward significance to higher Constructive Independent Play (*p* = 0.061). In the low social load condition, as compared to controls the SCT group showed higher Constructive Independent Play (*p* = 0.006). Lastly, in the high social load condition, as compared to controls, the SCT group showed lower Interaction behaviors (*p* < 0.001), higher Constructive Independent Play (*p* = 0.015) and lower No Response scores (*p* < 0.001; see Fig. 2).

### Social Impairments Associated with Autism Spectrum Disorders

#### Social Impairments in SCT vs. Controls

**Fig. 3:**
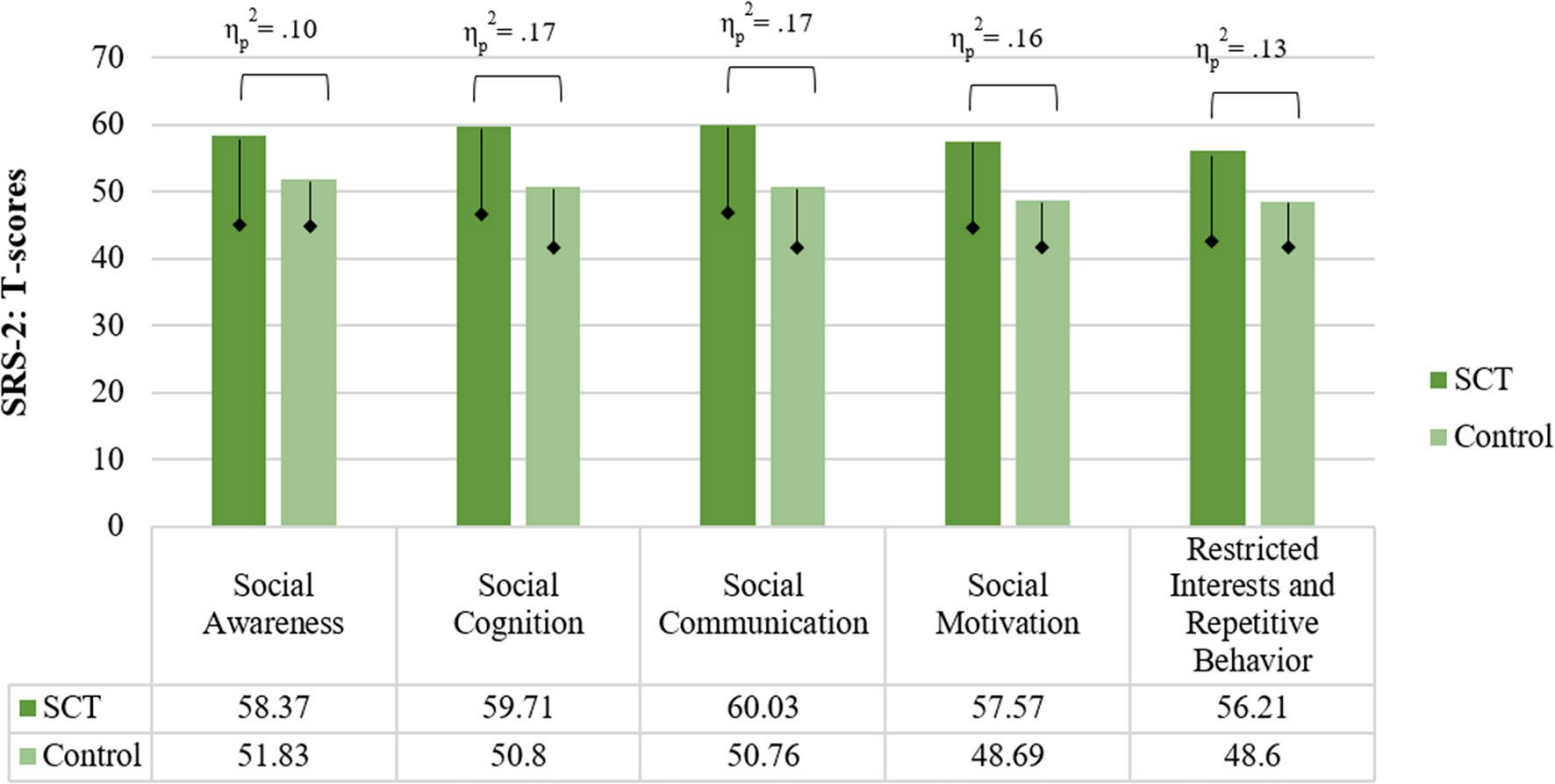
Social impairments associated with Autism Spectrum Disorder (ASD) in the SCT and control group (Mean). η_p_^2^ = effect size; = Standard Deviation (only lower bar depicted)

Scores on the SRS differed between the SCT and control group for all SRS subscales (*F* (5,134) = 11.87, *p* < 0.001, η_p_^2^ = 0.31). These results indicate that children with SCT have elevated social impairments as compared to their peers, on the domains of social awareness (SCT: *M* = 9.13*, SD* = 3.41; control: *M* = 7.34*, SD* = 2.58), social cognition (SCT: *M* = 10.99*, SD* = 5.30; control: *M* = 5.64*, SD* = 3.29), social communication (SCT: *M* = 18.44*, SD* = 9.28; control: *M* = 9.31*, SD* = 5.42), social motivation (SCT: *M* = 9.59*, SD* = 5.83; control: *M* = 5.13*, SD* = 2.97), and restricted interests and repetitive behaviors (SCT: *M* = 6.99*, SD* = 5.68; control: *M* = 2.93*, SD* = 2.84), with medium to large effect sizes. When evaluating total scores normalized for age and gender 72.9% of the SCT group scored in the average range, and 27.1% showed elevated ASD symptoms that are clinically significant: 15.7% of the children with SCT scores in the moderate range (T-score > 65 and < 75), 11.4% scores in the severe range (T-score < 75). See Fig. 3 for T-scores in the SCT and control group on domains of the SRS.

### Developmental Effects on Early Social Behavior

**Fig. 4:**
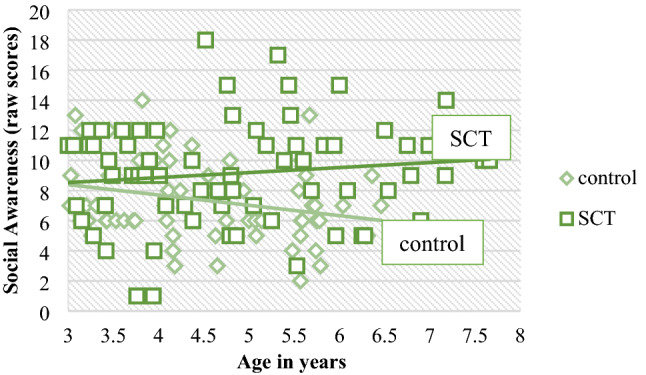
Moderating effect of age on social awareness; higher scores indicate difficulties in social awareness

In order to investigate the moderating effect of age on differences between the SCT and control group PROCESS analyses were carried out. Because aggressive behavior was almost non-existent in both the SCT and the control group during the ASIEP, and the distribution of aggression was insufficient, aggressive behavior was not included in the analyses. No moderating effects of age were found on social interaction behavior. See Table 2 for exact *B*, *t*- and *p*-values of the PROCESS models. Regarding social impairments, a moderating effect of age was found for social awareness (*p* = 0.028), indicating that the difference between children with SCT and control children was larger in older ages (see Fig. 4).

**Table 2:** PROCESS models of the moderating effect of age (group x age) on social interaction behavior and social impairments in the SCT and control group

Social interaction behavior (*N* = 188; age 1–7.5 years)		*B*	95% CI	*t*_***age***_	*p*_***age***_
No social load	Interaction	.31	− .15, .77	1.32	.188
	CIP	.33	− .42, 1.07	.87	.384
	No response	− .33	− .95, .28	− 1.07	.287
Low social load	Interaction	.40	− .25, 1.04	1.22	.225
	CIP	− .06	− .69, .56	− 0.20	.838
	No Response	− .24	− .85, .38	− 0.76	.450
High social load	Interaction	− .16	− .74, .43	− 0.53	.595
	CIP	− .02	− .50, .46	− 0.09	.929
	No Response	.11	− .21, .42	0.66	.507

Social Impairments (*N* = 140; age 3–7.5 years)					
Social awareness		− 1.02	− 1.93, − .11	− 2.22	.028
Social cognition		− .98	− 2.31, .36	− 1.45	.150
Social communication		− 2.01	− 4.30, .29	.09	.086
Social motivation		− .60	− 2.00, .81	− 0.84	.403
Restricted interests and repetitive behavior		− .91	− 2.26, .45	− 0.35	.188

*CIP* constructive independent play

### The Role of Global Cognitive and Language Level in Early Social Behavior

#### Structured Observations of Social Interactions

Behavioral outcomes on the ASIEP-3 (Interaction, Constructive Independent Play, No Response, Aggressive) were not correlated with global cognitive level and receptive language skills. However, total Interaction scores were positively correlated with expressive language skills (*r* = 0.142, *p* = 0.048). Therefore, expressive language skills were added as covariate in the group analyses. The overall differences between the SCT and control group remained significant, even when level of expressive language was added as covariate in the analysis, *F* (2.02, 659.21) = 6.97, *p* < 0.001, η_p_^2^ = 0.04. See Table 3 for descriptive statistics of global cognitive level, receptive and expressive language skills in the SCT and control group.

**Table 3:** Descriptive statistics in the SCT and control group: cognitive and language skills

	Age group	*N*	Missing	SCT	Control	SCT vs. control		
				*M (SD)*	*M (SD)*	*p* value	Group differences	Effect-size (η_p_^2^)
*Cognitive development* standard score; Bayley-III	1–2 years	33 SCT 30 control	0	99.55 (13.60)	99.71 (13.98)	.962		
*Receptive language development* scaled score; Bayley-III	1–2 years	33 SCT 30 control	0	9.24 (2.87)	12.23 (2.57)	< .001	SCT < control	.24
*Expressive language development* scaled score; Bayley-III	1–2 years	33 SCT 30 control	0	9.33 (3.20)	11.45 (2.85)	.007	SCT < control	.11
*Total IQ* standard score; WPPSI-III	3–7 years	64 SCT 71 control	5	95.28 (19.70)	108.24 (13.85)	< .001	SCT < control	.13
*Receptive language* standard score; PPVT-III	3–7 years	68 SCT 66 control	6	99.70 (15.01)	108.67 (12.44)	< .001	SCT < control	.10
*Expressive language* scaled score; CELF- Preschool	3–7 years	67 SCT 69 control	4	8.18 (3.05)	11.38 (2.55)	< .001	SCT < control	.25

*SCT* Sex Chromosome Trisomy

#### Social Impairments

Total scores on the SRS were negatively correlated with global cognitive functioning (*r* = − 0.520, *p* < 0.001) and expressive language skills (*r* = − 0.490, *p* < 0.001), but were not correlated with receptive language skills. Therefore, global cognitive functioning and expressive language skills were added as covariate in the group difference analyses. The difference on social impairments between the SCT and control group remained significant, even when global cognitive functioning and expressive language skills were added as covariates, *F* (5,125) = 4.19, *p* = 0.001, η_p_^2^ = 0.14.

In order to investigate whether social impairments were more pronounced in children with SCT with a below-average IQ, Total IQ is categorized into two groups (IQ < 84: below average; IQ > 85: average). The distribution of karyotypes (XXX, XXY, XYY) was similar between the two IQ groups (χ^2^ (2) = 0.29, *p* = 0.864). Two separate t-tests were carried out to investigate differences in social impairments between SCT and control children in both IQ-groups. In the average IQ-group, we found differences between the SCT (*M* = 49.70, *SD* = 21.10), and control children (*M* = 30.18, *SD* = 13,47; *t* (71,97) = 5.59, *p* < 0.001) with a large effect size (Cohens’ *d* = 1.10). Similar, in the below average IQ-group, we found differences between the SCT (*M* = 67.67, *SD* = 30.32), and control children (*M* = 34.33, *SD* = 5.13; *t* (18,66) = 4.31, *p* < 0.001), with a large effect size (Cohens’ *d* = 1.53). Based on these statistical tests and comparison of the effect sizes, these results indicate that both SCT children with average IQ as well as the SCT children with below average IQ have significantly increased social impairments, with relatively more severe social impairments in the below average IQ-group.

### Karyotype Differences Within the SCT Group

MANCOVAs were carried out in order to measure differences between the various karyotypes on social interaction behaviors and social impairments, accounting for the effect of age. For social interaction behavior, no differences between karyotypes were found, *F* (8,166) = 0.81, *p* = 0.597. A significant difference between karyotypes was found for social impairments, when accounting for the effect of age (*F* (10,126) = 2.37, *p* = 0.013, η_p_^2^ = 0.16). Across karyotypes, the XYY subgroup showed more pronounced social impairments in the domains of social cognition, social communication, and restricted interests and repetitive behavior, with medium to large effect sizes. Social awareness and motivation were similar across the three karyotypes. See Table 4 for exact *M, SDs, p* values and effect sizes.

**Table 4:** Differences in total social interaction behavior and social impairments across karyotypes (M, SD)

	**XXX**	**XXY**	**XYY**	*p* value	Group differences	Effect size (η_p_^2^)
Social interaction	*n* = 30	*n* = 44	*n* = 15			
Interaction	19.93 (7.24)	17.16 (7.29)	16.13 (10.12)	.478		
Constructive	20.00 (8.00)	22.93 (8.83)	21.27 (8.41)	.259		
Independent Play						
No response	7.93 (9.17)	7.73 (7.53)	10.20 (11.02)	.362		
Aggressive	0.00 (0.00)	0.11 (0.62)	0.20 (0.41)	.521		
Social Impairments	*n* = 27	*n* = 29	*n* = 14			
Social awareness	9.26 (3.15)	8.45 (3.28)	10.29 (4.05)	.300		
Social cognition	11.78 (4.91)	9.10 (4.26)	13.36 (6.80)	.037	XYY > XXY	.10
Social communication	18.78 (7.90)	14.55 (5.75)	25.86 (12.94)	.001	XYY > XXX/XXY	.20
Social motivation	10.70 (6.47)	8.03 (3.58)	10.64 (7.73)	.222		
Restricted interests and repetitive behavior	7.26 (4.64)	5.07 (4.18)	10.43 (8.35)	.019	XYY > XXY	.11

Higher scores on domains of Social Impairments indicate more impairments

### Recruitment Bias Within the SCT Group

Within the SCT group we tested with ANOVAs for differences on total scores between the three recruitment groups (prospective follow-up after prenatal diagnosis/information seeking parents/clinically referred cases), accounting for the effect of age. There were no significant differences for total social interaction behavior and social impairments associated with ASD, indicating that how children with SCT enrolled in the study was not related to their outcomes on social interaction behavior and social impairments. See Table 5 for exact *M, SDs* and *p *values*.*

**Table 5:** Differences in total social interaction scores and social impairments across recruitment groups (M, SD)

	Prospective follow-up	Information seeking parents	Clinically referred cases	*p* value
Social interaction	*n* = 49	*n* = 22	*n* = 18	
Total raw score	37.33 (14.23)	40.95 (16.09)	37.67 (12.36)	.426
Social impairments	*n* = 30	*n* = 22	*n* = 18	
Total raw score	51.00 (24.58)	56.55 (26.20)	60.83 (25.20)	.414

## Discussion

The current study adds to our understanding of the impact of SCT (XXX, XXY, XYY) on early social adaptive development in children aged 1 to 7.5 years. We evaluated profiles of social interactions in young children with SCT during structured behavior observations of a play situation, and vulnerability for social impairments that are associated with Autism Spectrum Disorder (ASD), based on daily life behaviors in a relatively large international sample.

Already early in life, an impact of SCT was found on the development of social interaction abilities: during a standardized behavior observation of a play situation, young children with SCT between the age of 1 and 7.5 years displayed on average less instances of social engagement as compared to their age matched peers, i.e. they showed less interaction behavior and more independent play. To illustrate, young children with SCT show a tendency to initiate and/or maintain less interactions, less imitations and mimics of play models, and to use less gestures and signs during communication, as compared to their age related peers. Also, young children with SCT more frequently demonstrate social withdrawal from interaction as evidenced by solo play. Aggressive or negative behaviors were almost non-existent in our study sample.

Interestingly, social interaction and withdrawn behavior differed as a function of social load: young children with SCT showed less social interactions when social load was absent, and more social withdrawal when social load was high, as compared to their peers. Social impairments were most pronounced when the level of social load was high, as children with SCT show less interactions and more severe social withdrawal (i.e. no observable or self-stimulating behavior) in the high social load condition. These findings suggest that social input and demands from the environment are conditional for the formation of social behavior in interaction with the social environment, as the results show that children with SCT may be able to shape social behavior when the environment provides little social input, but that they have difficulties with actively coping with varying levels of environmental social load and with responding to complex social information (i.e. information with a high social load). When extending the analysis to control for the potential impact of cognitive and language abilities on social behavior during high social load, these patterns remained significant.

These results fit with earlier studies that investigate the impact of the extra X- and Y-chromosome on social phenotypes in older age groups, showing that SCT on average is associated with shyness, social withdrawal, difficulties in peer-relationships, reduced social assertiveness, and communication difficulties (Bender et al., 1999; Geschwind & Dykens, 2004; Otter et al., 2010; van Rijn et al., 2014), and reported increased rick for mild symptoms of social anxiety (van Rijn et al, 2014; Wilson et al., 2019). Our findings add to the existing knowledge that an impact of SCT on social development can already be found during the first years of life, a developmental period in which social adaptive behavior rapidly develops as a function of brain maturation (Soto-Icaza et al., 2015), and that social abilities are dependent on the complexity of social information. Social interaction vulnerabilities showed to be independent of age and karyotype (XXX, XXY, XYY), which may suggest a stable impact of SCT during early development. As fewer positive social experiences and more social avoidance during early life can lead to less opportunities to develop social adaptive behavior (Beesdo et al., 2009), it is important to monitor and (preventively) support early social cognitive and behavioral development of children with SCT.

The current study showed that children with SCT are better able to attune their social behavior in interactions if social load is low. It may be that children with SCT have difficulty with selecting sensory information from their environment that in turn drives the challenges they face with producing social adaptive behavioral responses. It is known that sensory processing plays a major role in vulnerabilities on the social domain in children with neurobehavioral developmental conditions, such as Attention-Deficit/Hyperactivity Disorder and Autism Spectrum Disorder, suggesting that difficulties with processing sensory information play an important role in impairments in self-regulation and social adaptation (Cheung & Siu, 2009; Little et al., 2018; Sanz-Cervera et al., 2017). Basic sensory stimuli processing is fundamental in gaining access to social information from the environment and therefore in further shaping social behavior (Dellapiazza et al., 2021). Although difficulties in sensory processing are reported in individuals with SCT in clinical single case reports (Tartaglia et al., 2015), and the suggestion has been made earlier that rapid processing in auditory and other sensory modalities underlie language difficulties in adult men with 47, XXY (Geschwind et al., 2000), sensory profiles of individuals with SCT were not studied so far. It is therefore important to investigate the impact of SCT on profiles of sensory information processing on different sensory modalities, as for example on auditory and visual aspects of environmental stimuli.

The observed deficits in structured social interactions showed to be extend to impairments in daily life social behavior as reported by their parents, as we found that young children with SCT are at increased risk for social impairments associated with ASD. Within the SCT group, 27.1% had total social impairment scores in the clinical range (15.7% in the moderate clinical range and 11.4% in the severe clinical range). These findings are in line with earlier studies that investigated social impairments in individuals with SCT with broad age range groups from school age into adolescence (Cordeiro et al., 2012; Tartaglia et al, 2010; van Rijn et al., 2014; Wilson et al., 2019). The current study adds to the existing literature by showing an impact of the extra X and Y chromosome on ASD symptomatology that is present from a very young age. We found that in a small age range sample (3–7 years), social impairments already arise early in life, and are relatively stable during early development.

More specifically, across all karyotypes we found increased risk for significantly elevated social impairments for all the measured dimensions: social awareness, social cognition, social communication and social motivation. Young children with SCT also showed significantly more restricted interests and repetitive behaviors as compared to their age matched peers. When comparing overall effect sizes of the SCT vs. control differences on domains of social impairments, social awareness was an area of relative strength, including abilities such as being aware of certain social cues as for example facial expressions and body language. Earlier studies investigating the impact of SCT on social impairments found comparable effects: Tartaglia et al. (2010) found a relative strength in social awareness in boys with XXY, and Cordeiro et al. (2012) found mean scores for social motivation just above the normal range cutoff in boys with XXY and XYY. These results may suggest that children with SCT have relatively intact social awareness, which may be different from children with ASD, a reasoning that has already been suggested by van Rijn et al. (2014) and Wilson et al. (2019). These findings are in line with the results of the structured play observations in the current study with respect to social interaction behavior, showing that level of social input and demands from the environment indicates specific types of social deficits, and that social interaction behavior seems to be less affected when the social load from the environment was low. These results may suggest that young children with SCT are aware of their social environment, but are not able to adequately shape their behavior during social interactions.

However, in our sample with an age range of 3–7 years, we found age variability for impairments in the domain of social awareness, which were more affected in older children with SCT. This developmental effect could possibly account to a certain degree for the relative strength of social awareness and motivation we detected in young children with SCT. It is therefore important that future studies examining the impact of SCT on the social functioning and related psychopathology, use a developmental approach, given the detailed information it provides on the impact of SCT at different developmental stages of life. This knowledge can be used in developing specific age-related preventive intervention strategies for young children with SCT aimed to support social development.

Relative to SCT groups bearing an extra X-chromosome, we found a particularly pronounced vulnerability for social impairments in boys with XYY with regard to social cognition, social communication and restricted interests and repetitive behavior, but not for social awareness and social motivation. These findings suggest, on average, a more evident profile of social impairments in XYY associated with a typical ASD behavioral profile, and are consistent with earlier studies that compared social impairments across SCT karyotypes. For example, it was found that boys with XYY have a higher risk for clinical diagnoses of ASD, compared to boys and girls with an extra X-chromosome (Cordeiro et al., 2012; Ross et al., 2012; Tartaglia et al., 2017; Wilson et al., 2019).

Several neurocognitive mechanisms may underlie the difficulties in social interaction behavior under different conditions of social load, and risk on social impairments as observed in the current study. In the current study, we explored the role of global cognitive and language abilities in early social behavior (observed social interactions and daily life social impairments). First, for observed social interactions, we found that global cognitive level and receptive language skills were not associated with social interaction profiles. Although expressive language skills were correlated with level of social interactions, the ability of children to express themselves through verbal communication could not explain the difference between young children with SCT and their typically developing peers when it comes to social interaction behavior. Second, with regard to social impairments in daily life, these impairments showed to be correlated with global cognitive level and expressive language skills, but not with receptive language abilities. Also for social impairments, cognitive level and expressive language abilities could not explain the difference between the SCT and control group. Our results reveal that social impairments were found in both low IQ and average IQ groups, although particularly pronounced in children with SCT with a below average IQ. We can conclude that SCT has a specific impact on early social development, independent of the level of global understanding of the context and the ability to communicate with others in using language.

Our findings should be considered in light of several limitations. First, the majority of individuals with SCT still remains undetected during life (Berglund et al., 2019), although the group of diagnosed young children is rapidly growing with the introduction of the NIPT. In this study, social outcomes were not dependent on recruitment strategy (i.e. prospective follow-up group, information seeking parents group, or clinically referred cases group), which suggests that our findings are representative for this group of diagnosed children. However, it remains unsure to what degree the findings in this study can be generalized to those who have SCT, but remain undiagnosed. Second, although a considerable percentage of boys with XYY (Klinefelter Syndrome) received testosterone treatment (48%), we were unable to investigate the effect of testosterone treatment on social outcomes in the XXY group. Studies with suitable designs (randomized and placebo-controlled trials) could give insights into the effects of testosterone on behavioral outcomes in young boys with Klinefelter Syndrome.

Despite the above limitations, the current study with a relatively large and international sample of young children with SCT provides a more detailed understanding of the early impact of SCT on social adaptive development. Our findings reveal that difficulties with social interactions arise already early in life of children with SCT. We found that level of social load is associated with specific types of deficits, in terms of lower social interaction attempts and more withdrawn behavior that were most pronounced when the level of social load was high. These observed social vulnerabilities during structured interactions showed to extend to daily life, as we found increased levels of social impairments that are associated with ASD. These findings suggest that the extra X and Y chromosome impact social adaptive development from a very early age, and stresses the importance of early routine monitoring and (preventive) support of social development and risk on social impairments related to ASD in young children with SCT.
